# A Novel Inductive Displacement Sensor Based on Dual-Excitation and Single-Sensing Coils for Core Displacement Measurement

**DOI:** 10.3390/s25092827

**Published:** 2025-04-30

**Authors:** Longjiang Gao, Qiwei Xu, Yiru Miao, Wei Zhang, Chunlei Wang, Mengshu Li, Shihan Tang

**Affiliations:** 1 State Key Laboratory of Power Transmission Equipment Technology, School of Electrical Engineering, Chongqing University, Chongqing 400444, China; gaolongjiang7@cqu.edu.cn (L.G.); xuqw@cqu.edu.cn (Q.X.); miaoyiru@cqu.edu.cn (Y.M.); w_zhang@cqu.edu.cn (W.Z.); 2 Science and Technology on Reactor System Design Technology Laboratory, Nuclear Power Institute of China, Chengdu 610213, China; mslims@163.com (M.L.); tsh1994@126.com (S.T.)

**Keywords:** inductive displacement sensor, multi-group coil structure, high-precision, large-stroke, finite element simulation

## Abstract

This article develops a new inductive displacement sensor with a segmented multi-group coil structure, which is suitable for the displacement measurement of control rods in nuclear reactors. Each group coil of the sensor consists of two excitation coils and one sensing coil. The excitation and sensing coils are segmented to extend the linearity range of the displacement sensor. It abandons the traditional sensor’s method of using nonlinear compensation to achieve large-stroke displacement measurement. Providing an alternating current (AC) signal to the excitation coil and processing the induced voltage generated by each sensing coil can directly achieve the high-precision measurement of core displacement. The mathematical model of the variations in the sensing coil voltage caused by the movement of the core is established. The impacts of the excitation coil structure, the number of turns of the excitation coil, and the excitation frequency on the output characteristics of the designed sensor are analyzed by finite element simulation. Based on the analysis and design, a sensor prototype is built and tested in the laboratory. The measurement results show that the linearity error is 0.35% and the maximum measuring error can be limited within 1.5 mm, which is sufficient to meet the practical requirements in a nuclear reactor environment.

## 1. Introduction

A control rod is the only movable equipment in the pressure vessel of a nuclear reactor. Under normal conditions, the startup, shut-down, and reactivity control of the reactor are mainly achieved by adjusting the position of the control rod [1]. The accurate and reliable measurement of the control rod position is important for the safe operation of the reactor. Currently, the control rod displacement measurement sensor can be sorted into several types [2], including the inductance type, the capacitance type, the magnetostrictive type, the ultrasonic type, and the optical encoder type [3]. Among them, grating encoders have the advantages of large range, high precision, and nanometer resolution, and they are widely used in precision equipment for micro/nanometer measurement [4]. However, the grating encoder is susceptible to temperature, electromagnetic interference, and vibration [5]. Furthermore, control rod measurements do not require micrometer/nanometer levels and are therefore not applicable to nuclear reactor applications. Since inductive displacement sensors are characterized by robust anti-interference ability, corrosion resistance, low cost, and long service life [6,7], they are widely used in the field of nuclear reactors [8,9,10,11]. However, the main problem facing inductive displacement sensors is that the ratio of the sensor size to the linear range of the sensor is too large, and they are less precise for translational movement, limiting the applications of such a sensor [12]. With the advances in equipment manufacturing, various fields have put forward higher requirements on the measurement accuracy and range of inductive displacement sensors.

Many researchers have improved the sensor structure to extend the maximum linearization range under a limited sensor size. A structural design is adopted in which the number of winding turns is arranged in an exponential trend [13]. Although this method can extend the sensor’s linearization range, it is difficult to evenly wind the coil in an exponential form. The authors of [14,15] propose a differential variable reluctance transformer (DVRT), which is a structure similar to the linear voltage differential transformer (LVDT) without the secondary winding. The core position is calculated by recording the measured value of the voltage between the terminal and the center tap, which increases the sensor’s stroke length ratio. However, its output linearity has not been improved so far. A transformer-based inductive displacement sensor was proposed in [16,17]. This study presented a sensor operating at 100 Hz, achieving a linearity error of 1.6% over a full 400 mm stroke. The influence of temperature was investigated, revealing that the temperature of the iron rod had a negligible effect on the induced voltage of the sensor, while the temperature of the aluminum cylinder had highest impact on the induced voltage.

Some researchers have established a new sensor model and improved the linearity within a limited stroke length through simulation optimization. The study in [18] established a fractional-order sensor model, which could increase stroke length without compromising sensitivity and linearity. However, the design of the fractional-order sensor required a large number of capacitors and resistors, resulting in a complex and large circuit structure. A mathematical model based on a multi-objective evolutionary algorithm was proposed in [19]. Although it could design a sensor with high linearity, the full stroke of the designed sensor was relatively small and unsuitable for a large-stroke displacement sensor. Several techniques have been suggested in the literature to overcome the challenge of a short measurement range caused by the nonlinear response characteristics of inductive displacement sensors, but these are tedious and time-consuming.

The above-mentioned studies are all about improving the sensor’s stroke length under limited sensor size. Regarding sensor measurement accuracy, the relevant studies have used the DSP algorithm [20], numerical calculations [21], signal circuit processing [22,23], and neural network algorithms [24,25] to improve the measurement accuracy of sensors. Among them, neural network algorithms are the most widely used. An adaptive calibration technique for displacement measurement based on a neural network algorithm was proposed in [26]. This measurement technique produced an output that was linear for the full scale of the input range, making the output adaptive to variations in the structural parameters of the sensor, excitation frequency, and ambient temperature. A two-stage functional link artificial neural network (FLANN) algorithm was proposed. The inverse model was found to have higher precision when using a best-fit model of the measured data, which enabled the sensor to realize high-precision displacement measurement. Non-dominated sorting genetic algorithms (NSGAs)-II and -III have been utilized to optimize geometry configuration by adjusting the inner parameters corresponding to the dimensions of the interior components of a sensor [27], with the aim of improving measurement accuracy. Although the above-mentioned methods can improve measurement accuracy, the use of artificial neural network technology requires a high-speed signal processor to calculate the adaptive neural network model, which increases the computational burden of the hardware [28].

Therefore, in order to develop a high-precision inductive displacement sensor suitable for large-stroke displacement measurement, a novel sensor design with a segmented multi-group coil structure is proposed in this paper. The measurement principle of the designed sensor involves providing an AC signal to the excitation coils and processing the induced voltage generated by the sensing coil to directly achieve the measurement of core displacement. The main contributions of this paper are summarized as follows:

(1) A novel inductive displacement sensor with a segmented multi-group coil structure is proposed, which utilizes the optim linear range of the sensing coil’s voltage of to measure the core displacement; the stroke range can be easily increased.
(2) Mathematical and finite element simulation models are established to systematically investigate the influence of sensor coil structure, number of turns, and excitation frequency on output characteristics through theoretical analysis and simulation studies.
(3) A sensor prototype is built to test its performance. The experimental results demonstrate that the measurement accuracy of the designed sensor is less than 1.5 mm and linearity error is 0.35%. The measurement accuracy is greatly improved compared to the other inductive displacement sensors used in nuclear reactors.

The remainder of this paper is organized as follows: Section 2 introduces the structure design and measurement principle of the sensor in detail. The relationship between the sensor coil structure, number of turns, and excitation frequency and the sensor output characteristics is simulated and analyzed in Section 3. Section 4 introduces the experiment conducted to test the sensor prototype and discusses the experimental results. Section 5 presents the conclusions.

## 2. Design of Sensor

### 2.1. Structure and Measurement Principle of Sensor

**Figure 1:**
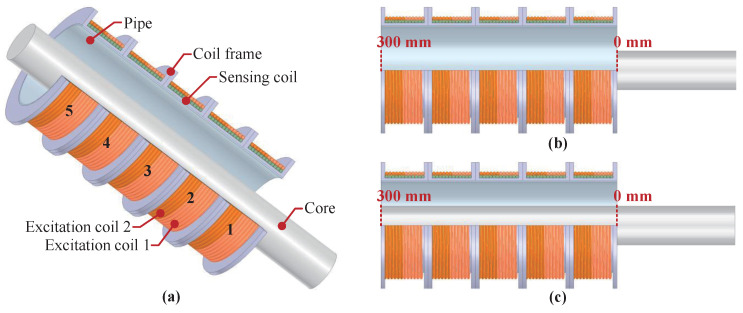
Structure of the designed sensor. (**a**) Structural schematic diagram; (**b**) Sensor without the core; (**c**) Sensor with a fully-inserted core.

The structural diagram of the inductive displacement sensor proposed in this paper is shown in Figure 1a. Figure 1b,c show the core at different positions. The mechanical structure of the sensor is mainly composed of excitation and sensing coils, the coil frame, and the pipe. The designed sensor has five segmented coils for the excitation and sensing systems; each segmented coil includes two excitation coils, a sensing coil, and a coil frame. The pipe is utilized for coil frame fixation. The sensing coil is wound around the coil frame, and the two excitation coils are connected in parallel outside the sensing coils. The excitation coils of the designed sensor structure can also be connected in series. To prove the performance advantages of the parallel structure sensor, this paper simulates and experimentally analyzes the sensors of the two structures at the same time. Compared with the traditional three-coil inductive displacement sensor (LVDT) [25,29], the designed sensor adopts a stacked winding of the coil, so the overall size is relatively small although the stroke is the same. In addition, the excitation coils and the sensing coil are independent of each other, making the sensor output signal more easily processable. In addition, by increasing the number of coil segments, the stroke length of the sensor can also be increased.

**Figure 2:**
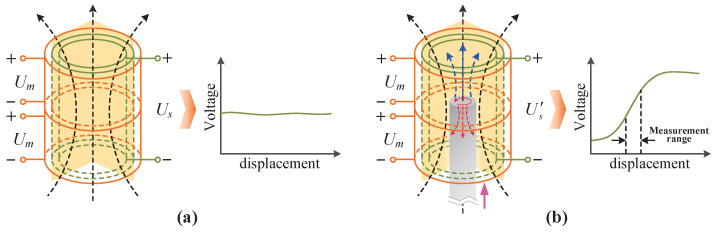
Measurement principle of the designed sensor: (**a**) The core does not enter the coil. (**b**) The core enters the coil.

To illustrate the measurement principle, a single segmented coil is used as an example, as depicted in Figure 2. When an excitation voltage is supplied to the excitation coils, the sensing coil will generate an induced voltage. The magnetic field inside the excitation coils at this time is shown in Figure 2a. It can be seen that the sensing coil fully receives the magnetic flux generated by the excitation coils and generates a constant voltage. When the core is close to and enters the sensor, variations in the sensing coil voltage are as shown in Figure 2b. The core generates a magnetization effect and an eddy current effect according to the magnetic permeability and conductivity under the alternating magnetic field. The magnetization effect is dominant compared with the eddy current effect due to the high relative permeability of the core, which leads to an increase in the coil’s total magnetic flux. The magnetic flux will increase as the length of the core entering the coil increases until saturation. Thus, the inductance of the excitation coils and the induced voltage in the sensing coil change with the displacement of the core. The displacement information of the core can be accurately calculated by detecting the sensing coil’s voltage signal following signal processing.

Traditional inductive displacement sensors use changes in the displacement of the core to cause changes in the inductance of the excitation coil, converting the inductance into a voltage or current to calculate the core displacement. Compared with the traditional methods, the displacement calculation method proposed in this paper eliminates the need for redundant conversion circuit design, making it easy to implement and reducing the cost of the sensor system.

### 2.2. Mathematical Model of Sensor

**Figure 3:**
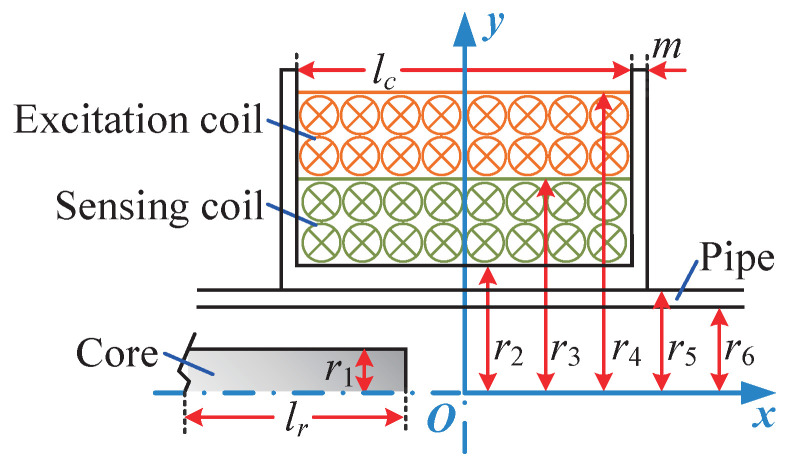
Mathematical model of sensor.

The coil of the inductive displacement sensor is generally made of multiple circular current-carrying wires wound together. By integrating the magnetic induction intensity of the circular current-carrying wire along the axial direction of the coil, we can obtain the magnetic induction intensity model of a single-layer cylindrical coil. To obtain the mathetical model for the magnetic induction intensity model of multi-layer cylindrical coils, however, it is necessary to integrate along the thickness direction of the coil. As shown in Figure 3, in order to analyze the specific factors that affect the output characteristics of the designed sensor, a mathematical model is established for single-segmented coils.

When the excitation coil is supplied with an AC signal and the core does not enter the coil, the magnetic flux expression generated by the exciting coil can be expressed as follows:

(1) $$\Phi_{e}=\frac{L_{c}I}{N_{1}}=\frac{L_{c}U_{m}\sin\left(2\pi f_{m}t\right)}{N_{1}\sqrt{R_{c}^{2}+{\left(2\pi f_{m}L_{c}\right)}^{2}}}$$

where $U_{m}$ is the excitation voltage amplitude; $f_{m}$ is the excitation frequency; *I* is the current in the excitation coil; $N_{1}$ is the turn number of the excitation coil; and $R_{c}$ and $L_{c}$ are the equivalent resistance and equivalent inductance of the excitation coil when the core does not enter the coil.

From Equation (1), the magnetic flux $\Phi_{s}$ in the sensing coil and the induced voltage $U_{s}$ of the sensing coil when the core does not enter the coil can be expressed as follows:

(2) $$\Phi_{s}=\lambda \frac{L_{c}I}{N_{1}}=\lambda \frac{L_{c}U_{m}\sin\left(2\pi f_{m}t\right)}{N_{1}\sqrt{R_{c}^{2}+{\left(2\pi f_{m}L_{c}\right)}^{2}}}$$

(3) $$U_{s}=-N_{2}\frac{d\Phi_{s}}{dt}=\lambda \frac{2\pi f_{m}N_{2}L_{c}U_{m}\cos\left(2\pi f_{m}t\right)}{N_{1}\sqrt{R_{c}^{2}+{\left(2\pi f_{m}L_{c}\right)}^{2}}}$$

where λ is the coupling coefficient of the magnetic flux of the excitation coils in the sensing coils, which is determined by the coil manufacturing craft; and $N_{2}$ is the turn number of the sensing coil.

When the core is close to and subsequently enters the sensor, the core will be magnetized and generate a magnetic field with an enhanced effect. It should be noted that due to the core moving slowly, the effect of the core speed on the sensor output is neglected here. Therefore, the induced voltage generated by the sensing coil varying with the displacement of the core can be expressed as follows [30]:

(4) $$U_{s}^{\prime }=\frac{N_{1}N_{2}\left(\mu_{r}-1\right)\mu_{0}\pi^{3}r_{3}^{2}r_{1}^{2}l_{r}f_{m}KU_{m}\sin\left(2\pi f_{m}t\right)}{l_{c}\left(r_{4}-r_{3}\right)\sqrt{R_{c}^{2}+{\left(2\pi f_{m}L_{c}\right)}^{2}}}$$

in which

(5) $$K=\left(x+\frac{l_{c}}{2}\right)ln\frac{r_{2}+\sqrt{r_{2}^{2}+{\left(x+\frac{l_{c}}{2}\right)}^{2}}}{r_{1}+\sqrt{r_{1}^{2}+{\left(x+\frac{l_{c}}{2}\right)}^{2}}}-\left(x-\frac{l_{c}}{2}\right)ln\frac{r_{2}+\sqrt{r_{2}^{2}+{\left(x-\frac{l_{c}}{2}\right)}^{2}}}{r_{1}+\sqrt{r_{1}^{2}+{\left(x-\frac{l_{c}}{2}\right)}^{2}}}$$

where $\mu_{r}$ is the magnetic permeability of the core; $\mu_{0}$ is the magnetic permeability of air; and *x* is the distance from the center of the coil to the center of the moving core. Be combining Equations (3) and (4), the sensor sensitivity *S* can be derived, as shown in Equation (6).

(6) $$S=\frac{\Delta U}{\Delta x}=\frac{{U^{\prime }}_{s}-U_{s}}{\Delta x}$$

where ΔU is the change in sensing coil voltage; and Δx refers to the position variation. From Equation (6), it can be concluded that, when the core moves a unit distance, the larger the voltage change ΔU, the higher the sensitivity *S* of the sensor. It also means that the measurement accuracy will be higher. From the above analysis, we can conclude that the factors affecting the sensing coil voltage increment are as follows:

(1) External excitation source: excitation frequency $f_{m}$ and excitation voltage amplitude $U_{m}$;
(2) Sensor structure: coil equivalent resistance $R_{c}$, equivalent inductance $L_{c}$, number of turns of the excitation coil $N_{1}$, and the sensing coil $N_{2}$.

The influence of the excitation voltage amplitude $U_{m}$ on the sensing coil voltage is monotonous based on the theoretical analysis. The larger the excitation voltage amplitude, the larger the sensing coil voltage; however, the sensing coil voltage increment will not change significantly.To improve the sensitivity of the sensor, we use the finite element method (FEM) to study the relationships between the coil structure, number of coil turns, excitation frequency, and sensor output characteristics.

## 3. Finite Element Analysis

Since 3D analysis is more time-consuming and needs more memory, a 2D axisymmetric finite element method is used for the modeling. Considering the complexity of the manufacturing process and the overall structural size, the length of a single excitation coil is selected to be 25 mm, and the length of the sensing coil is 50 mm. As the excitation voltage amplitude is restricted by the current density that the wire can withstand, excessive current amplitude will cause the coils to heat up, which will have a detrimental effect on measurement accuracy. So, the wire diameter of 0.31 mm is selected, as indicated in a previous study [31]. The main simulation parameters are shown in Table 1. The pipe and the coil frame in this article are made of bakelite, which has the characteristics of insulation and nonmagnetic conductivity. The non-magnetic properties of bakelite cause it to have no effect on the electromagnetic field in ANSYS 2022R1 simulation, so the material properties and structural parameters do not affect the simulation and experimental results.

### 3.1. Excitation Coil Structure Analysis

#### 3.1.1. Series and Parallel Structure Analysis

**Figure 4:**
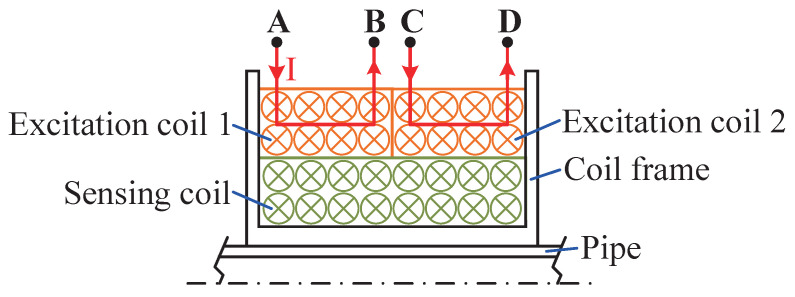
Different arrangements of excitation coils (i.e., in series and in parallel).

The dual-excitation coils in the designed sensor have two winding structures. The different arrangements of the excitation coils in series and in parallel are shown in Figure 4. When the excitation coils are connected in series, B and C are connected. At this time, the direction of the current passing through the coil is A–B–C–D. When the excitation coils are connected in parallel, A and C are connected to the positive pole, and B and D are connected to the negative pole. The directions of the current passing through the two excitation coils are A–B and C–D, respectively.

**Figure 5:**
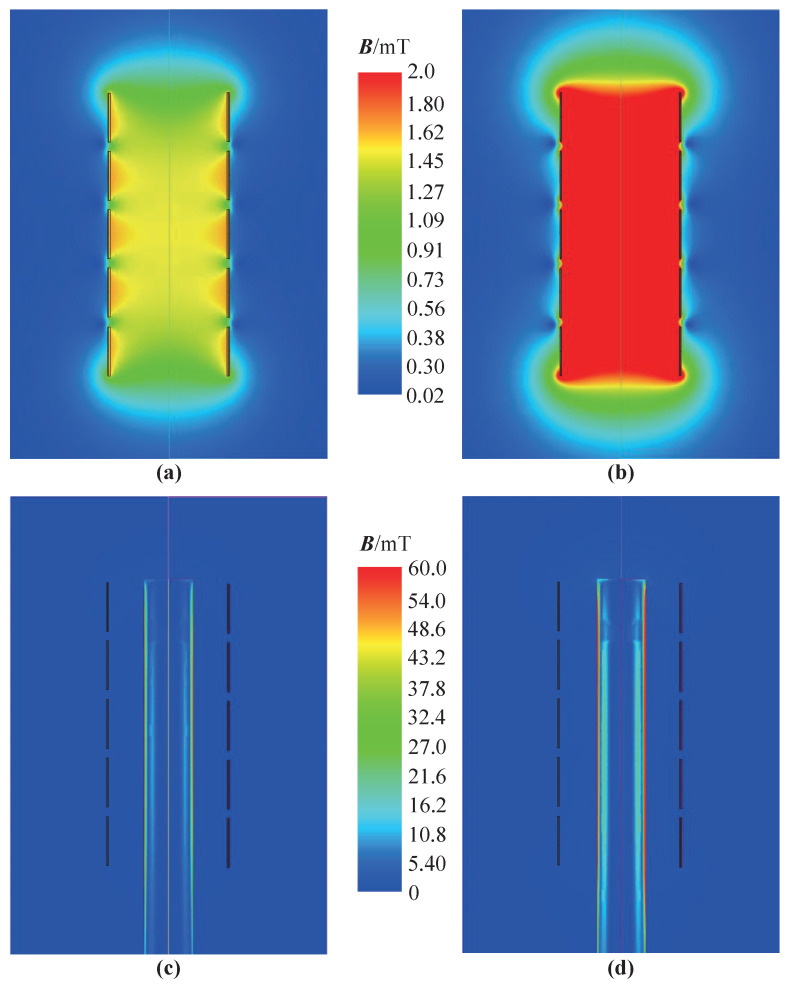
Magnetic field distribution of the excitation coils in series and parallel: (**a**) Two excitation coils in series without the core. (**b**) Two excitation coils in parallel without the core. (**c**) Two excitation coils in series with a fully inserted core. (**d**) Two excitation coils in parallel with a fully inserted core.

In this paper, the magnetic field analysis of the sensor with two winding structures is carried out. The simulation results, as shown in Figure 5, indicate the magnetic field distribution of the designed sensor without the core and with a fully inserted core. The magnetic field distributions of the excitation coils in series and in parallel, when the sensor is without the core, are shown in Figure 5a and Figure 5b, respectively. Using a magnetic induction intensity reference value of 2 mT, the simulation results indicate that that the magnetic induction intensity in the detection area is significantly higher than the that of the excitation coils in series. The maximum magnetic induction intensity of the detection area increases from 1.92 mT to 3.92 mT, more than two times. The magnetic field distribution of the excitation coils in series and in parallel, when the sensor has a fully inserted core, are shown in Figure 5c and Figure 5d, respectively. The magnetization effect of the core in the detection area is more obvious, which increases the sensing coil voltage amplitude caused by the core when the excitation coils are in parallel.

Figure 5b,d show the magnetic field simulation results when the excitation coils are connected in parallel and the excitation current is in the same direction. It is observed that the magnetic field in the center of the coil is enhanced when in the same direction as the current, resulting in an improvement in the overall magnetic field inside the sensor. When the core moves inside the sensor, the magnetic flux caused by the core changes greatly, resulting in a large change in the sensing coil voltage, which is beneficial to the displacement measurement of the core. However, when the excitation currents are in opposite directions—the current directions are A–B and C–D—the magnetic field in the center of the coil is in the opposite direction and is weakened, resulting in a weaker magnetic field distribution inside the sensor. When the magnetic core moves inside the sensor, the magnetic flux change caused by the core is small, resulting in a small change in the sensing coil voltage, which is not conducive to the displacement measurement of the core. The analysis of the simulation results indicates that using a parallel winding structure with the same current direction is suitable for improving magnetic field induction intensity. The subsequent analysis will focus on the excitation coils in a parallel configuration.

#### 3.1.2. Analysis of Asymmetry in Excitation Coils

**Figure 6:**
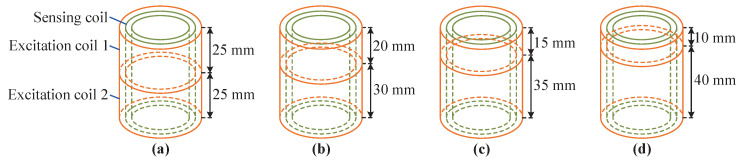
Schematic diagram of different lengths of excitation coils: (**a**) 25 mm:25 mm. (**b**) 20 mm:30 mm. (**c**) 15 mm:35 mm. (**d**) 10 mm:40 mm.

The ANSYS 2022 R1 software is used to establish a sensor model under the symmetrical and asymmetrical conditions of two excitation coils. The length diagrams of the two excitation coils are shown in Figure 6a–d. The lengths of the two excitation coils are 25 mm/25 mm, 20 mm/30 mm, 25 mm/35 mm and 10 mm/40 mm, respectively. The length of the sensing coil remains unchanged. A dynamic simulation analysis is carried out on the established simulation model, and the core is made to rise from the bottom to the top at a step length of 5 mm. The sensing coil voltage simulation data are processed using MATLAB 2023b software. It can be concluded that when the two excitation coils are symmetrical, the sensor has the highest sensitivity, which is 0.067 V/m. When the lengths of the two excitation coils are 20 mm/30 mm, 15 mm/35 mm, and 10 mm/40 mm, the sensitivity of the sensor is 0.0654 V/mm, 0.0639 V/mm, and 0.0626 V/mm, respectively. It means that as the asymmetry of the two excitation coils increases, the sensitivity of the sensor gradually decreases. Therefore, in order to obtain maximum sensor sensitivity, the two excitation coils need to remain symmetrical.

### 3.2. Influence of the Number of Turns of the Excitation Coil on Sensor Performance

**Figure 7:**
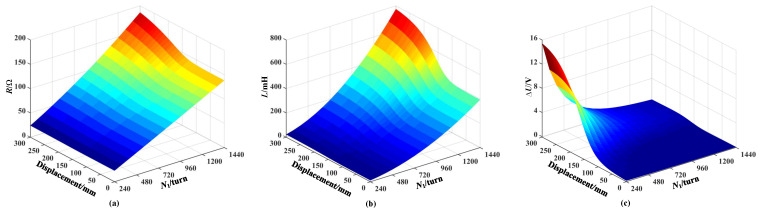
Influence of the number of turns in the excitation coil for coil number 3: (**a**) Resistance. (**b**) Inductance. (**c**) Sensing voltage increment.

**Figure 8:**
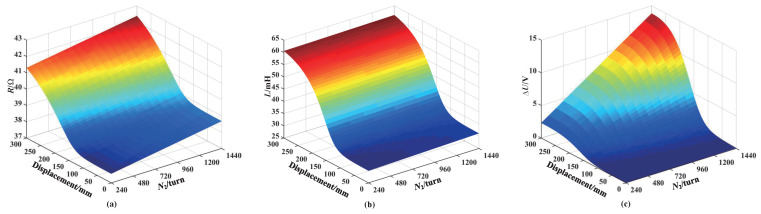
Influence of the number of turns in the sensing coil for coil number 3: (**a**) Resistance. (**b**) Inductance. (**c**) Sensing voltage increment.

**Figure 9:**
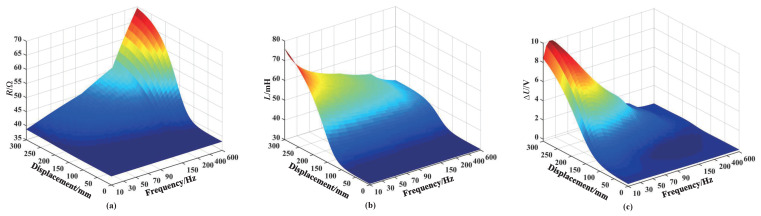
Influence of the excitation frequency: (**a**) Resistance. (**b**) Inductance. (**c**) Sensing voltage increment.

When the number of excitation coil turns are different, the alternating magnetic field formed by the excitation coil has different magnetization and eddy current effects on the core, thereby affecting the induced electromotive force output by the sensing coil. In order to study the influence of the excitation coil turns on the output characteristics of the sensor, a simulation is conducted on the excitation coil resistance, inductance, and the sensing coil voltage increment when the core enters the sensor under different excitation coil turns, as shown in Figure 7. Here, the voltage increment ΔU represents the voltage variation in sensing coil number 3, while the displacement denotes the position of the core within the sensor (refer to Figure 1a). The movement range of the core is 0–300 mm. It is important to note that variations in the inductance and resistance of the excitation coil, as depicted in Figure 7, Figure 8 and Figure 9, are analyzed based on the excitation coil located to the right of coil number 3 (refer to Figure 1b).

The resistance and inductance of the excitation coil increase with the increase in the number of coil turns, as shown in Figure 7a and Figure 7b, respectively. In addition, the resistance and inductance increase as the length of the core entering the sensor increases. This is because the eddy currents generated in the core consume energy from the coil, and the resistance of the coil increases. In addition, the magnetization effect of the core is dominant compared with the eddy current effect due to its high relative permeability, leading to an increase in coil inductance. As the number of turns in the excitation coil increases, the coil impedance rises, causing a reduction in both the primary magnetic field produced by the excitation source and the additional magnetic field generated by the core, thereby decreasing the overall magnetic field. Therefore, the sensing coil voltage increment decreases as the number of excitation coil turns increases, as shown in Figure 7c. The sensing coil voltage increment increases as the length of the core entering the sensor increases, which is consistent with the theoretical analysis of the measurement principle mentioned above. Based on the above-mentioned analysis, the number of turns of the excitation coil should be as small as possible.

### 3.3. Influence of the Number of Turns of the Sensing Coil on Sensor Performance

Figure 8 shows the simulation results of the excitation coil resistance and inductance, as well as the sensing coil voltage increment, when the core enters the sensor under a different number of sensing coil turns. From Figure 8a,b, it can be seen that the excitation coil resistance and inductance increase slowly with the increase in the number of sensing coil turns. This is because the excitation coil is wound around the outside of the sensing coil. Under the condition that the inner diameter of the number of sensing coil and excitation coil turns remain unchanged, the increase in the number of sensing coil turns means that the inner and outer diameters of the excitation coil will also increase, resulting in an increase in the resistance and inductance of the excitation coil. Additionally, the excitation coil resistance and inductance increase with the length of the core entering the sensor increases; the reasons of this outcome have been explained in the previous section. The sensing coil voltage increment increases as the number of sensing coil turns increases, and the voltage increment increases as the length of the core entering the sensor increases, as shown in Figure 8c. In order to obtain a larger sensing coil voltage increment, the number turns of the sensing coil should be as large as possible, but the difficulty of the coil winding process should also be considered.

### 3.4. Influence of the Excitation Frequency on Sensor Performance

For inductive displacement sensors, the excitation frequency is also one of the key factors affecting the performance of the sensor. Figure 9 shows the simulation results of the excitation coil resistance and inductance, as well as the sensing coil voltage increment, when the core enters the sensor under different excitation frequencies. The excitation coil resistance increases with the increase in excitation frequency, while the inductance decreases with the increase in excitation frequency, as shown in Figure 9a,b. It is evident that the variations in coil resistance and inductance with frequency are small. This is because for the core, the magnetization effect in the coil is dominant. Figure 9c illustrates the influence of different excitation frequencies on the voltage of sensing coil number 3 as the sensor transitions from a no core state to a fully inserted core state. It can be observed that the lower frequency makes the voltage variation generated by the core more obvious. When the core reaches the center position of sensing coil number 3 at 150 mm, the voltage increment attains its maximum value of 3.063 V at an excitation frequency of 40 Hz, compared to the other frequencies. We assume the operating range of sensing coil number 3 is defined within [120 mm, 180 mm] (150 mm ± 30 mm). Within this operating range, a significant enhancement in voltage increment is observed when employing excitation frequencies within [35 Hz, 55 Hz]. This study employs the closed-loop control of a single-phase inverter to generate AC excitation voltage, given that the control design for 50 Hz single-phase inverters has been extensively researched and documented in the literature. On the other hand, considering the overall performance of the sensor, the excitation frequency can only be selected as a compromise between the sensitivity and the response time. We selected 50 Hz as the excitation frequency to achieve optimal performance of the AC excitation voltage.

## 4. Experimental Verification

### 4.1. Measurement Setup

**Figure 10:**
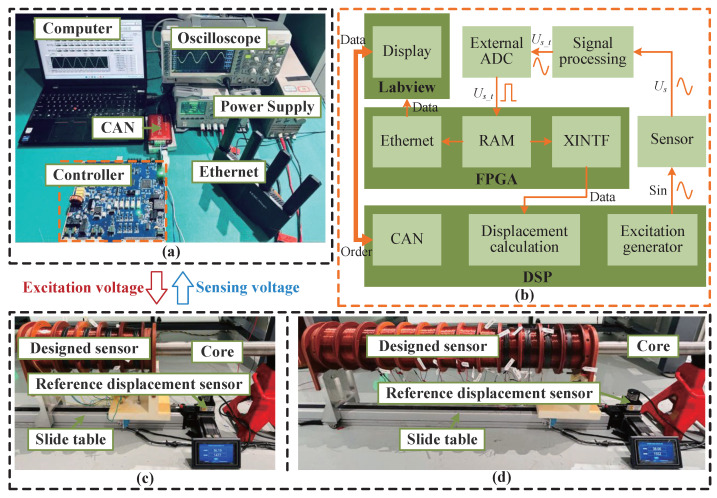
Experimental setup: (**a**) Experimental platform. (**b**) Controller circuit schematic diagram. (**c**) Designed sensor prototype (short stroke). (**d**) Designed sensor prototype (large stroke).

The sensor prototype parameters and simulation model parameters are almost the same, as shown in Table 1. Therefore, the sensor experimental prototype is fabricated, and the experimental platform is constructed, as shown in Figure 10. It mainly consists of the following: computer, controller, oscilloscope, power supply, CAN, reference displacement sensor, slide table, core, and designed sensor. The computer with LabVIEW 8.5 software is used to display the excitation voltage signal, sensing voltage signals, and core displacement in real time. The absolute displacement sensor is used as a reference displacement measurement sensor to measure the core position. The slide table is used to move the sensor, which changes the core position in the sensor.

In Figure 10b, the controller is mainly composed of the following four modules: excitation module, signal processing circuit, FPGA data communication module, and DSP operation control module. A single-phase inverter with a closed-loop control circuit is designed as the excitation module to supply power to the excitation coil of the designed sensor, with all units operated simultaneously. The induced voltage analog signals produced by the sensing coil are high noisy, affecting the calculation of the core displacement. So, it is necessary to transform the analog signal into a digital signal through a low-pass filter, reduction, and external analog-to-digital converter (ADC) sampling chip in the signal processing circuit. The FPGA chip EP4CE22F17I7N (Intel, Santa Clara, CA, USA) is employed to store the sampled digital signal data in RAM and subsequently output the data to the DSP through the XINTF bus. In addition, it can transmit data to the LabVIEW 8.5 for display through ethernet communication. The DSP chip TMS320F28335 (TI, Dallas, TX, USA) reads the data sent by the FPGA, calculates the core displacement in real time through algorithm processing, and communicates with LabVIEW 8.5 through the CAN. Furthermore, it also accepts orders from LabVIEW 8.5, such as the adjustment of excitation voltage amplitude and frequency according to the order.

Figure 10c,d present schematic diagrams of short-stroke and large-stroke sensors, respectively. It is observed that the displacement sensor proposed in this article can increase the stroke length by increasing the number of segments of coils, which has the advantages of being simple to implement and efficient operation.

### 4.2. Static Characteristics Test

**Figure 11:**
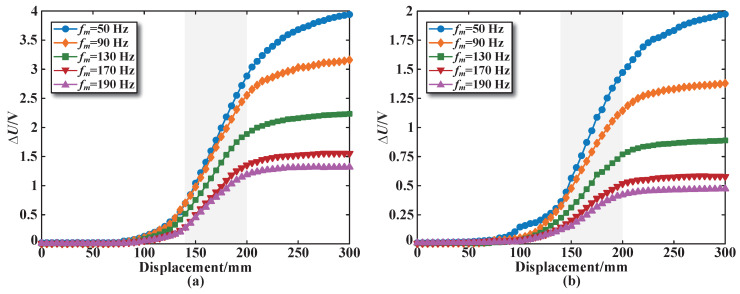
The static characteristics curve for sensing coil number 3: (**a**) Parallel structure. (**b**) Series structure.

The static characteristics test of the segmented coils in the designed sensor is carried out through the experimental platform shown in Figure 10. Taking sensing coil number 3 in the sensor as an example, Figure 11a shows the change curve representing the sensing coil voltage increment under the condition that the core enters the coil. The displacement range measured is from 0 mm to 300 mm for the core (refer to Figure 1b), and the arithmetic mean of the sensing coil voltage increment is obtained through three repeated independent experiments. In addition, a comparative analysis of the sensing coil voltage increment for the sensor with the excitation coils arranged in series is carried out, and the experimental results are shown in Figure 11b.

As shown in Figure 11, the internal magnetic field of the coil varies with the movement of the core, leading to changes in the induced electromotive force of the sensing coil. Regarding the stroke of the sensor, the larger the length of the core entering the coil, the higher the sensing coil voltage, demonstrating a nonlinear relationship. It is clear that the center position of sensing coil 3 shows a linear change within a range of about ±30 mm around 150 mm. However, in order to obtain higher measurement accuracy, it is necessary to perform linear analysis on the operating range data near the center position of 150 mm. The specific operating range selection principle is shown in Section 4.4. Finally, [140 mm, 200 mm] is selected as the linear operating range of sensing coil 3, as shown in the gray area in Figure 11. From the comparison of Figure 11a,b, it is evident that under identical excitation conditions, the sensing coil voltage increment for the parallel structure of the excitation coil is greater than that for the series structure, approximately twice as much. This means that for every unit distance the core moves, the output variation of the designed sensor will be larger, significantly enhancing its sensitivity.

**Figure 12:**
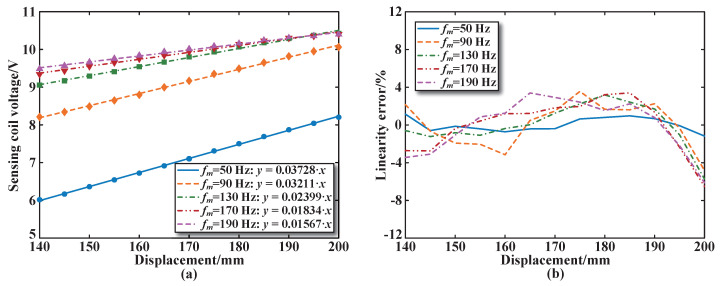
Performance of excitation coils in parallel at different frequencies: (**a**) Calculated sensitivity. (**b**) Calculated linearity error.

**Figure 13:**
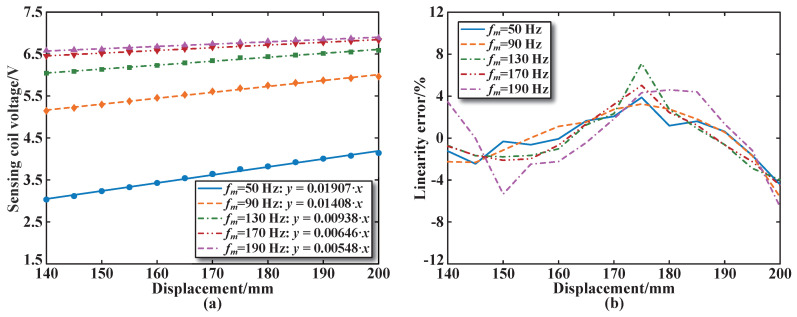
Performance of excitation coils in series at different frequencies: (**a**) Calculated sensitivity. (**b**) Calculated linearity error.

Figure 12 and Figure 13 show the experimental results, fitting calculation results, and linearity errors of the sensing coil voltage for sensing coil number 3 at different frequencies for both the parallel and series arrangements of the sensor excitation coils. Figure 12 shows that when the excitation frequency is 50 Hz, the maximum sensitivity of the sensor with the parallel structure is 0.03728 V/mm, and the maximum linearity error is less than 1.2%, which is smaller than that of the other excitation frequencies. Figure 13 shows that the sensor sensitivity in the series structure is 0.01907 V/mm at an excitation frequency of 50 Hz, which is lower than that of the parallel structure. The maximum linearity error is less than 4.5%, which is also smaller than that of other excitation frequencies.

### 4.3. Dynamic Characteristics Test

**Figure 14:**
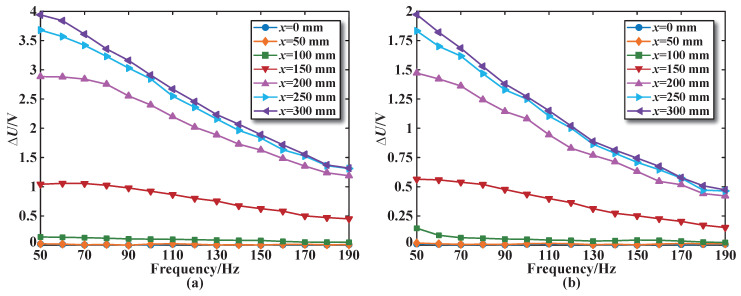
The dynamic characteristics curve for sensing coil number 3: (**a**) Parallel structure. (**b**) Series structure.

The dynamic characteristics test of the segmented coils in the designed sensor is carried out. Figure 14a shows the change curve representing the sensing coil voltage increment at different excitation frequencies. The excitation frequency test range is 50–190 Hz, and the arithmetic mean of the output voltage increment is obtained by three repeated independent experiments. Similarly, the sensing coil voltage increment is compared for the sensor with a series structure of the excitation coils, as shown in Figure 14b.

From Figure 14, it can be seen that within the excitation frequency test range, when the core approaches and passes through the coil (x = 150 mm, x = 200 mm, x = 250 mm, and x = 300 mm), the sensing coil voltage increment decreases as the excitation frequency increases. When the core is far away from the coil (x = 0 mm, x = 50 mm and x = 100 mm), the change in the excitation frequency has almost no effect on the sensing coil voltage increment. This is because the magnetization effect generated by the core has almost no effect on the coil, which is equivalent to a coil in a no core state.

In summary, the overall trend of the experimental results is consistent with the simulation results, which proves the effectiveness of the sensor structure proposed in this paper.

### 4.4. Performance Test

The sensing coil outputs a quadrature amplitude modulation signal when the excitation signal is a sinusoidal AC, so the coherent demodulation method is required to restore the modulated signal to the sensing signal induced by the core. In order to simplify the signal detection process, the discrete Fourier transform (DFT) method is used to collect the sine wave peak value of the sensing coil voltage signal of the designed sensor. According to the application field of the designed sensor, the proposed DFT amplitude extraction algorithm and first-order, low-pass filtering algorithm are well suited for applications involving slowly varying or quasi-static displacements. These algorithms can significantly enhance resolution and stability in static or near-static conditions by reducing sensitivity to transient noise, allowing the sensor to obtain stable and reliable measurement results.

**Figure 15:**
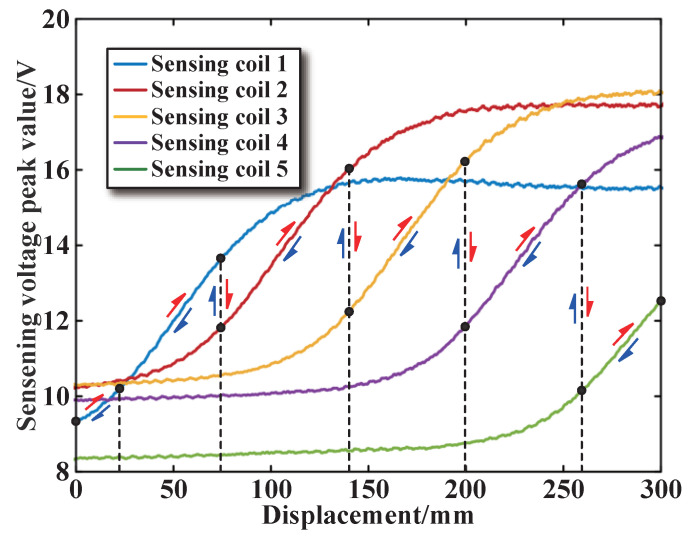
DFT sampling results of voltage peaks.

By connecting the collected peak values, we can obtain the change curve representing the sine wave peak values, which can reflect the changes in core displacement. Since the sensor proposed in this article contains five segmented coils, five sensing coil voltage peak curves can be obtained, and the experimental results are shown in Figure 15.

As shown in Figure 15, each segment has its corresponding core displacement measurement range. The five measurement ranges are connected end to end to measure the displacement of the core within the full stroke. For the selection of the linear operating range of the sensor, the linearity error and sensitivity of the operating range of each segment are used as the selection criteria. Taking the first segment as an example, according to the core displacement and sensing coil voltage peak data obtained from the experiment, the linearity error and sensitivity within different operating ranges can be calculated using MATLAB 2023b software, as shown in Table 2. From Table 2, it can be concluded that the first segment has the highest linearity error and the greatest sensitivity in the operating range [20 mm, 80 mm]. The linearity error and sensitivity are 0.47% and 0.0682 V/mm, respectively. We select this range as the optimal operating range of the first segment of coils. Similarly, when the excitation coils are connected in series, the sensor sensitivity is about 0.0336 V/mm. When the excitation coils are connected in parallel, the sensitivity of the sensor is about twice that of the series structure, which is consistent with the simulation analysis.

The optimal operating range of the remaining four segments can be obtained using the above-mentioned method. It should be noted that the linear operating range for each segmented coil is not fixed at 60 mm. The operating range needs to be determined based on the linearity error and sensitivity calculations of the experimental data, so that the five operating ranges constitute the entire measurement range. It should be noted that coil 1 has two core displacement measurement ranges ([0 mm, 20 mm] and [20 mm, 80 mm]) as the characteristics deviate from linear at the bottom end of the sensor. For an actual control rod position indicator in nuclear reactors, the control rod stroke is always shorter than the sensor. The first measurement range can be ignored, but in order to improve the overall measurement accuracy of the designed sensor, this paper performs polynomial fitting on the first measurement range.

#### 4.4.1. Accuracy Test

**Figure 16:**
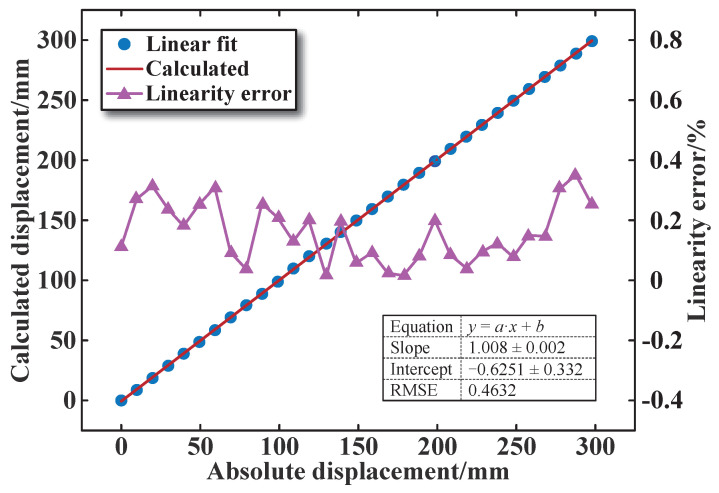
Linearity error versus core displacement.

Figure 16 shows the fitting curve of the relationship between the calculated core displacement values and the absolute displacement values. It can be seen that the calculated displacement values are almost equal to the absolute displacement values. The root mean square error (RMSE) and linearity error in the full range (0–300 mm) are 0.4632 and 0.35%, respectively. This also proves that the test results of the designed sensor are relatively stable, and the calculated and absolute displacement values exhibit a good linear relationship.

#### 4.4.2. Repeatability Tests

**Figure 17:**
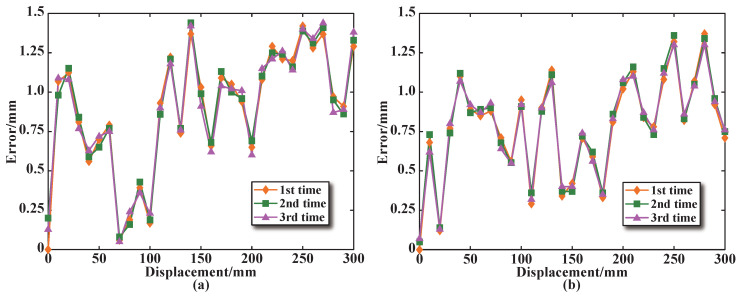
Repeatability of sensor: (**a**) Positive repeatability within full range (0–300 mm). (**b**) Negative repeatability within full range (0–300 mm).

Further, the repeatability experiments of the designed sensor are carried out in measuring steps of 10 mm, with two mechanical cycles over the range of (0–300 mm), and three measurement repetitions for assessing the positive and negative repeatabilities, respectively. As shown in Figure 17, the positive and negative repeatability errors are both within 1.5 mm, which proves that the designed sensor has high measurement accuracy. Table 3 compares the main performance parameters of the sensor we designed with those of the other inductive displacement sensors used in nuclear reactors. The linearity error of shown in a previous study [8] in Table 3 is lower than that of the sensor designed in this paper, as the range in that study [8] is much larger than the range of the designed sensor. Within the same measurement range, using the relative error calculation method, it can be seen that the linearity error of the designed sensor is much smaller than that obtained in the previous study [8]. This paper only tests the measurement accuracy of the designed sensor within the measurement range of 0–300 mm. The method of increasing the stroke is shown in Figure 10c. The size of the stroke is determined by the number of the segmented coils added.

All experiments were conducted at room temperature in this paper. The temperature can change the relative magnetic permeability of the core but has little effect on sensor sensitivity [17]. In a nuclear reactor environment, the material of the control rod is completely different from that of the core. The influence of temperature on the sensor may be caused by changes in the special magnetic material properties of the core with temperature. At the same time, the sensor heats up the coil when it is in operation, causing the coil temperature to rise. We measured the temperature increase caused by coil heating. However, compared with the high temperature environment in a nuclear reactor, the temperature effect caused by coil heating is negligible. It should be noted that the working environment in the nuclear reactor is not of a constant temperature. Usually, the temperature at the bottom of the sensor is much lower than the temperature at the top, which has an impact on the detection voltage of the sensor. Given the limitations of our experimental conditions, it is currently impossible to simulate the actual working environment of a nuclear reactor in such a short time.

## 5. Conclusions

In this paper, a novel structure with dual excitation coils and a single sensing coil is proposed for core displacement measurement, which is suitable for the displacement measurement of control rods in nuclear reactors. Firstly, the structural design and measurement principle of the designed displacement sensor are introduced. Then, the influence of the structure, number of turns, and excitation frequency of the sensor excitation coils on the sensing coil and the sensor output characteristics is simulated and analyzed. Finally, the tests are conducted to verify the measurement performance of the designed displacement sensor. The following conclusions are drawn:

(1) The coil structure and parameters are closely related to sensor output. For excitation coils with the same number of turns, the parallel structure enables a sensor output voltage increment that is approximately twice that of the series structure. For the sensing coil, the larger the number of turns, the greater the coil inductance and resistance, resulting in a larger sensing coil voltage increment, thereby improving sensor sensitivity.
(2) The excitation frequency has a great influence on the displacement measurement performance of the sensor. The excitation frequency and the output of the sensor show a nonlinear relationship. Within the range of 35 Hz–55 Hz, the sensing coil voltage increment is large. In order to facilitate the design of a single-phase inverter excitation circuit, the excitation frequency is selected as 50 Hz in this paper.
(3) After the experimental performance tests, the designed sensor is shown to have stable core displacement measurement performance, with a linearity error of 0.35% within the full 300 mm stroke. It can perform high-precision measurements of <1.5 mm, which meets the actual use requirements of a nuclear reactor.

The sensor designed in this paper can not only be used to measure the displacement of control rods in nuclear reactors but also the position of pistons in pneumatic aluminum cylinders. By keeping the sensor structure unchanged while changing the sensor size and number of coil turns, it can be applied to industrial applications such as linear valve control in chemical pipelines and the axial displacement monitoring of steam turbines. 

## Figures and Tables

**Table 1:** Simulation parameters of the designed sensor.

Parts	Parameters	Value
Excitation soure	Amplitude ($U_{m}$)	10 V
	Frequency ($f_{m}$)	50 Hz
Sensing coil	Number of turns for the sensing coil ($N_{2}$)	800
	Length ($l_{c}$)	50 mm
	Inner radius ($r_{2}$)	60 mm
	Outer radius ($r_{3}$)	61.2 mm
Excitation coils 1 and 2	Number of turns for the excitation coil ($N_{1}$)	400
	Length ($l_{c}/2$)	25 mm
	Inner radius ($r_{3}$)	61.2 mm
	Outer radius ($r_{4}$)	62.4 mm
Coil frame	Coil spacing (*m*)	5 mm
	Inner radius ($r_{5}$)	57 mm
	Outer radius ($r_{2}$)	60 mm
Pipe	Inner radius ($r_{6}$)	55.5 mm
	Outer radius ($r_{5}$)	57 mm
Core	Length ($l_{r}$)	1000 mm
	Radius ($r_{1}$)	25 mm

**Table 2:** Linearity error and sensitivity in different operating ranges.

Operating Range	Linearity Error	Sensitivity
[10 mm, 70 mm]	1.38%	0.0639 V/mm
[15 mm, 75 mm]	0.82%	0.0671 V/mm
[20 mm, 80 mm]	0.47%	0.0682 V/mm
[25 mm, 85 mm]	0.77%	0.0674 V/mm
[30 mm, 90 mm]	1.17%	0.0647 V/mm
[35 mm, 95 mm]	1.39%	0.0618 V/mm
[40 mm, 100 mm]	1.48%	0.0582 V/mm

**Table 3:** Performance comparison between different inductive displacement sensors.

Performance Parameters	Ref. [8]	Ref. [9]	Refs. [10,11]	Designed Sensor
Measurement range	0–3571.9 mm	0–3571.9 mm	0–4238.6 mm	0–300 mm
Relative error	0.45%	1.7%	0.89%	0.5%

## Data Availability

The data presented in this study are available on request from the corresponding author.
